# The role of semantic abstractness and perceptual category in processing speech accompanied by gestures

**DOI:** 10.3389/fnbeh.2013.00181

**Published:** 2013-12-18

**Authors:** Arne Nagels, Anjan Chatterjee, Tilo Kircher, Benjamin Straube

**Affiliations:** ^1^Department of Psychiatry and Psychotherapy, Philipps-University MarburgMarburg, Germany; ^2^Department of Neurology and the Center for Cognitive Neuroscience, The University of PennsylvaniaPhiladelphia, PA, USA

**Keywords:** iconic gestures, deictic gestures, metaphoric gestures, functional magnetic resonance imaging, speech-associated gestures, cognition

## Abstract

Space and shape are distinct perceptual categories. In language, perceptual information can also be used to describe abstract semantic concepts like a “rising income” (space) or a “square personality” (shape). Despite being inherently concrete, co-speech gestures depicting space and shape can accompany concrete or abstract utterances. Here, we investigated the way that abstractness influences the neural processing of the perceptual categories of space and shape in gestures. Thus, we tested the hypothesis that the neural processing of perceptual categories is highly dependent on language context. In a two-factorial design, we investigated the neural basis for the processing of gestures containing shape (SH) and spatial information (SP) when accompanying concrete (c) or abstract (a) verbal utterances. During fMRI data acquisition participants were presented with short video clips of the four conditions (cSP, aSP, cSH, aSH) while performing an independent control task. Abstract (a) as opposed to concrete (c) utterances activated temporal lobes bilaterally and the left inferior frontal gyrus (IFG) for both shape-related (SH) and space-related (SP) utterances. An interaction of perceptual category and semantic abstractness in a more anterior part of the left IFG and inferior part of the posterior temporal lobe (pTL) indicates that abstractness strongly influenced the neural processing of space and shape information. Despite the concrete visual input of co-speech gestures in all conditions, space and shape information is processed differently depending on the semantic abstractness of its linguistic context.

## Introduction

In face-to-face communication people often use gestures to complement the content of their verbal message. People produce different kinds of gestures (McNeill, 1992), such as iconic gestures illustrating shape (e.g., “The ball is round”) or deictic gestures referring to spatial information in our physical environment (e.g., “The cat is sitting on the roof”; pointing gesture). Shape gestures resemble the information they convey, as when someone draws a circle in the air to indicate a round shape (“The table in the kitchen is round,” circle gesture). Space and shape gestures typically refer to concrete entities in the world. However, they can also make abstract references depending on the nature of the verbal message (McNeill, 1992; McNeill et al., 1993a,b). For instance, shape-related gestures can illustrate a deep connection between twins when the speaker touches the fingertips of both hands (“The twins had a spiritual bond between them”). Similarly space-related gestures can refer to abstract relationships or locations such as lifting the hand when saying that the discussion occurred at a very “high level.”

In direct face-to-face communication people use gestures (Ozyurek and Kelly, 2007), regardless of whether the utterances are concrete or abstract. In line with theories suggesting gestures may represent the phylogenetic origin of human speech (Corballis, 2003, 2009, 2010; Gentilucci and Corballis, 2006; Gentilucci et al., 2006; Bernardis et al., 2008), gestures might represent the basis of spatial or action representations in human language [for example, see Tettamanti and Moro (2011)]. Such spatial elements transferred into speech and gestures could be an expression of how our language is rooted in embodied experiences (Gibbs, 1996; Lakoff, 1987). Following this idea perceptual elements and the sensory-motor system might both contribute to the processing and comprehension of figurative abstract language (particularly in the context of metaphors such as “grasp an idea”), as suggested by the embodiment theory (Gallese and Lakoff, 2005; Arbib, 2008; Fischer and Zwaan, 2008; D'Ausilio et al., 2009; Pulvermüller and Fadiga, 2010). Thus, the investigation of the neural substrates underlying the processing of perceptual categories such as shape or space in the context of concrete vs. abstract language semantics would give an answer to this hypothesis.

Recent fMRI investigations have focused on the processing of speech and gesture for different gesture types beat gestures: (Hubbard et al., 2009); iconic gestures: (Willems et al., 2007, 2009); and metaphoric gestures: (Kircher et al., 2009; Straube et al., 2009, 2011a). In general, left hemispheric posterior temporal (Holle et al., 2008, 2010; Green et al., 2009) and inferior frontal brain regions (Willems et al., 2007; Kircher et al., 2009; Straube et al., 2009, 2011a) are commonly found for the semantic processing of speech and gesture. The left posterior temporal lobe (pTL) seems to be involved during the apprehension of co-verbal gestures, whereas the left inferior frontal gyrus (IFG) seems to be additionally recruited when processing gestures in an abstract sentence context (Kircher et al., 2009; Straube et al., 2011a, 2013) or when accompanying incongruent (“The fisherman has caught a huge fish,” while the actor is angling his arms) concrete speech (Willems et al., 2007; Green et al., 2009; Willems et al., 2009). However, these studies do not examine the neural effects of processing of concrete or abstract utterances with different perceptual categories, such as gestures referring to shape (e.g., “The ball is round”) or space (e.g., “The shed is next to the building”).

In a previous study, we compared brain activation in response to object-related (non-social) and person-related (social) co-verbal gestures (Straube et al., 2010). Person-related as opposed to object-related gestures activated anterior brain regions including the medial and bilateral frontal cortex as well as the temporal lobes. These data indicate that dependent of speech and gesture content (person-related vs. object-related) different brain regions are activated during comprehension. However, in the aforementioned study the content of the verbal utterances was confounded by differences in the level of abstractness, since person-related gestures are not only social, but also more abstract symbolic than object-related gestures (e.g., “The actor did a good job in the play”). Therefore, the specific influence of person-related and object-related content independent of abstractness was not disentangled.

Beside this evidence for a posterior to anterior gradient of processing for concrete to abstract speech-gesture information, it is generally assumed that specific regions of the brain are specialized for the processing of specific kinds of contents (Patterson et al., 2007). Information about shapes of objects are processed in lateral occipital and inferior temporal brain areas (e.g., Kourtzi and Kanwisher, 2000; Grill-Spector et al., 2001; Kourtzi and Kanwisher, 2001; Kourtzi et al., 2003; Panis et al., 2008; Karnath et al., 2009, whereas the parietal lobe is involved in processing of spatial information (Rizzolatti et al., 1997, 2006; Koshino et al., 2000, 2005; Rizzolatti and Matelli, 2003; Chica et al., 2011; Gillebert et al., 2011). Although gestures can be distinguished by perceptual category [e.g., deictic gestures convey spatial information and iconic gestures predominantly convey shape information (McNeill, 1992)] there is insufficient knowledge about the neural processing of these different perceptual categories in the context of abstract and concrete sentence contexts.

Here we investigate the way in which perceptual category and semantic abstractness of co-verbal gestures interact. Our experiment aims at the question whether different perceptual categories are processed in the same or in distinct brain regions, irrespective of their linguistic abstractness. To approach this research question, we applied a naturalistic approach comparing shape-related and space-related gestures in the context of concrete and abstract sentences.

On a cognitive level (concrete physical) gesture content has to be aligned with the content of speech, regardless of whether the message is concrete or abstract. We hypothesize that the effort to incorporate both abstract speech with concrete gestures will likely result in enhanced neural responses in the left inferior frontal cortex (Willems et al., 2007) and in bilateral temporal brain regions (Kircher et al., 2009) as compared to the concrete conditions, independent of perceptual category. With regard to shape-related and space-related gestural information we expected differential activation within the inferior temporal and parietal lobe, respectively. For the interaction of perceptual (space and shape) and semantic category (concreteness and abstractness) two alternative results were hypothesized: (1) If the same neural processes are engaged when processing shape and space information regardless of the abstractness of the message, we will find no significant activation in interaction analyses. In this case, conjunction analyses (e.g., aSP > aSH ∩ cSP > cSH) will result in common activation patterns in the parietal cortex for space and inferior temporal cortex for shape. (2) If abstractness influences the processing of shape-related and space-related gesture information, interaction analyses will show differential activations between conditions. Here, we expected an interaction since language content may differentially influence the interpretation of perceptual categories and consequently the neural processing predominantly in the left IFG and pTL. Enhanced neural responses in classical “language regions” would strengthen the assumption that perceptual categories are differentially processed if embedded into an abstract vs. concrete language context.

## Materials and methods

### Participants

Seventeen male right handed (Oldfield, 1971) healthy volunteers, all native speakers of German (mean age = 23.8 ± 2.7 years, range: 20–30 years, mean years of school education = 12.65 ± 0.86, range: 10–13 years), without impairments of vision or hearing, participated in the study. None of the participants had any serious medical, neurological or psychiatric illness, past or present. All participants gave written informed consent and were paid 20 Euro for participation. The study was approved by the local ethics committee. Because of technical problems one fMRI-data set was excluded from the analyses.

### Stimulus construction

A set of 388 short video clips depicting an actor was initially created, consisting of 231 concrete and 157 abstract sentences, each accompanied by co-verbal gestures.

Iconic gestures refer to the concrete content of sentences, whereas metaphoric gestures illustrate abstract information in sentences. For example in the sentences “To get down to business” (drop of the hand) or “The politician builds a bridge to the next topic” (depicting an arch with the hand), abstract information is illustrated using metaphoric gestures. By contrast, the same gestures can be iconic (drop of the right hand or depicting an arch with the right hand) with the sentences “The man goes down the hill” or “There is a bridge over the river” when they illustrate concrete physical features of the world. Thus, concrete utterances are those containing referents that are perceptible to the senses (“The man ascends to the top of the mountain”). Abstract sentences, on the other hand, contain referents that are not directly perceptible (“The man ascends to the top of the company”), where the spatial or shape terms in the utterance are being used figuratively. For the distinction between concrete and abstract concepts see Holmes and Rundle (1985).

Here we were interested in the neural processing of the following types of sentences accompanied by gestures: (1) utterances with concrete content and space-related perceptual information (cSP; “deictic gesture”); (2) utterances with concrete content and shape-related perceptual information (cSH; “iconic gesture”); (3) utterances with an abstract content and space-related perceptual information (aSP; “abstract deictic gestures”); and (4) utterances with an abstract content and shape-related perceptual information (aSH; “metaphoric gestures”).

All sentences accompanying gestures had a length of 5–10 words, with an average duration of 2.37 s (*SD* = 0.35) and a similar grammatical form (subject—predicate—object). The speech and gestures were performed by the same male actor in a natural, spontaneous way. This procedure was continuously supervised by two of the authors (Benjamin Straube, Tilo Kircher) and timed digitally. All video clips had the same length of 5 s with at least 0.5 s before and after the sentence onset and offset, respectively, where the actor did not speak or move.

### Stimulus selection: rating / material selection/matching

For stimulus validation, 17 raters not participating in the fMRI study evaluated each video on a scale ranging from 1 to 7 (1 = very low to 7 = very high) according to three content dimensions (space, shape and action information) and familiarity. Other general parameters like “understandability” and “naturalness” were previously validated and controlled for (for detailed information see (Green et al., 2009; Kircher et al., 2009; Straube et al., 2011a,b).

Material was selected to address our manipulations of interest (cf. above):

1. cSP = **C**oncrete content and **SP**ace-related information
2. cSH = **C**oncrete content and **SH**ape-related information
3. aSP = **A**bstract content and **SP**ace-related information
4. aSH = **A**bstract content and **SH**ape-related information

For each condition 30 sentences were selected to differentiate both factors. Therefore, co-verbal gestures conveying space-related perceptual information (cSP, aSP) were selected to have similar spatial rating scores independent of the level of the abstractness of the utterance (c vs. a). Abstract co-verbal gestures (aSP, aSH) were selected to be similarly abstract independent of the perceptual category of information (space or shape; see Table 1).

**Table 1:** **Experimental manipulations**.

**Condition**	**Space-related**				**Abstract**				**Shape-related**			
	**Mean**	***SD***	**95% confidence interval for mean**		**Mean**	***SD***	**95% confidence interval for mean**		**Mean**	***SD***	**95% confidence interval for mean**	
cSP	5.71	0.60	5.49	5.93	2.18	0.82	1.87	2.49	2.97	0.97	2.60	3.33
cSH	3.91	0.91	3.57	4.25	2.44	0.78	2.15	2.74	5.17	1.39	4.65	5.69
aSP	5.40	0.53	5.20	5.59	4.19	0.90	3.85	4.53	3.25	0.87	2.92	3.57
aSH	4.08	0.86	3.76	4.41	3.90	1.00	3.53	4.27	4.90	0.95	4.54	5.25
Total	4.78	1.08	4.58	4.97	3.18	1.24	2.95	3.40	4.07	1.44	3.81	4.33

*Rating results for the conditions cSP, cSH, aSP, and aSH for space-relatedness, abstractness and shape-relatedness*.

To confirm that our stimuli met our design criteria, we calculated analyses of variances for the factors perceptual (space-, shape related) and semantic category (concrete, abstract) as represented in the 2 × 2 experimental design.

As intended we found for the rating of spatial information a significant main effect for perceptual category [SP > SH; *F*_(1, 116)_ = 72.532, *p* < 0.001], but no significant effects for the main effect of semantic category [a vs. c; *F*_(1, 116)_ = 0.149, *p* = 0.603] or the interaction of perceptual and semantic category [*F*_(1, 116)_ = 3.250, *p* = 0.074].

For the rating of shape information we obtained again a significant main effect for perceptual category [SH > SP; *F*_(1, 120)_ = 98.466, *p* < 0.001], but no significant effects for the main effect of abstractness [a vs. c; *F*_(1, 120)_ = 0.001, *p* = 0.988] or the interaction of perceptual category and abstractness [*F*_(1, 120)_ = 2.053, *p* = 0.155].

For the rating of abstractness we obtained a significant main effect for abstractness [a > c; *F*_(1, 116)_ = 116.124, *p* < 0.001], but no significant effects for the main effect of perceptual category [SP vs. SH; *F*_(1, 116)_ = 0.005, *p* = 0.942] or the interaction of perceptual category and abstractness [*F*_(1, 116)_ = 2.975, *p* = 0.087]. For means and confidence intervals see Table 1. Together, these analyses confirm that stimulus selection worked out and stimulus characteristics for each condition met our design criteria.

For the control variables familiarity, naturalness and action information we found no significant main effects or interactions (for all *p* > 0.10). However, we found significant effects for understandability [main effect perceptual category: SP > SH: *F*_(1, 120)_ < 4.960, *p* = 0.028; interaction: *F*_(1, 116)_ < 17.704, *p* < 0.001], speech duration [main effect abstractness: a > c: *F*_(1, 116)_ = 9.024, *p* < 0.003] and gesture duration [main effect abstractness: c > a: *F*_(1, 116)_ < 10.821, *p* < 0.001]. However, differences in understandability were small (<0.22 rating points) and most likely because of ceiling effects in the aSP (skewness = −1.68; kurtosis = 4.31) and cSH (skewness = −1.40; kurtosis =1.80) conditions. For means and confidence intervals of the control variables see Table 2.

**Table 2:** **Control variables: understandability, familiarity and naturalness**.

**Condition**	**Understandability**				**Familiarity**				**Naturalness**			
	**Mean**	***SD***	**95% confidence interval for mean**		**Mean**	***SD***	**95% confidence interval for mean**		**Mean**	***SD***	**95% confidence interval for mean**	
cSP	6.63	0.21	6.55	6.71	4.98	0.97	4.62	5.34	4.87	0.48	4.69	5.05
cSH	6.84	0.14	6.79	6.89	4.75	0.77	4.46	5.04	4.97	0.48	4.79	5.15
aSP	6.82	0.17	6.75	6.88	5.10	0.91	4.76	5.44	4.80	0.59	4.58	5.02
aSH	6.75	0.20	6.68	6.83	4.80	0.89	4.47	5.13	4.80	0.49	4.62	4.98
Total	6.76	0.20	6.73	6.80	4.91	0.89	4.75	5.07	4.86	0.51	4.77	4.95

*Rating results for the conditions cSP, cSH, aSP, and aSH for the dimensions understandability, familiarity and naturalness*.

In the event-related fMRI study design focusing on the co-occurrence of speech and gesture, differences in speech or gesture duration should not have a crucial impact on our results. However, we included differences in speech and gesture duration for each event as a covariate of no interest in our single-subject design matrix.

Apart from the aforementioned factors, further differences in movement characteristics were found between the conditions. For all four conditions predominantly right (cSP = 19; cSH = 13; aSP = 16; aSH = 11) or bimanual movements were performed (cSP = 11; cSH = 17; aSP = 14; aSH = 19). To ensure that none of the patterns of neural activation were produced by differences in hand movements (right hand vs. both hands) and speech length, a separate control analysis was run accounting for the aforementioned dimensions. A set of 11 exactly paired video clips for each condition was used for the additional analysis.

To account for differences in the size of movements between conditions, we coded each video clip with regard to the extent of the hand movement. We divided the video screen into small rectangles that corresponded to the gesture space described by McNeill (1992); McNeill (2005) and counted the number of rectangles in which gesture movements occurred see Straube et al. (2011a). For each video the number of rectangles was also included as covariate of no interest in the single subject model.

### Experimental design and procedure

During the fMRI scanning procedure, videos were presented via MR-compatible video goggles (VisuaStim^©^, Resonance Technology, Inc.) and non-magnetic headphones (audio presenting systems for stereophonic stimuli: Commander; Resonance Technology, Inc.), which additionally dampened scanner noise.

**Figure 1:**
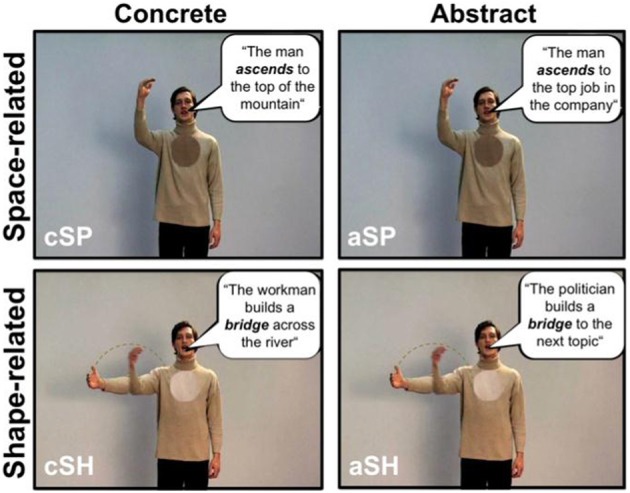
**Examples of the different speech and gesture video-clips**. The stimulus material consisted of video clips of an actor performing either space-related **(top)** or shape-related **(bottom)** gestures to corresponding sentences with an concrete **(left)** or abstract content **(right)**. One screen shot of an example video is shown for each condition (cSP, concrete space-related; cSH, concrete shape-related; aSP, abstract space-related; aSH, abstract shape-related). In order to exemplify the stimulus material German sentences are translated into English, and written in speech bubbles for illustration (unlike in the actual stimuli).

Thirty items of each of the four conditions were presented in an event-related design, in a pseudo-randomized order and counterbalanced across subjects. Each video was followed by a baseline condition (gray background with a fixation cross) with a variable duration of 3750–6750 ms (average: 5000 ms) see Figure 1.

During scanning participants were instructed to watch the videos and to indicate via left hand key presses at the beginning of each video whether the spot displayed on the actor's sweater was light or dark colored. This task was chosen to focus participants' attention on the middle of the screen and enabled us to investigate implicit speech and gesture processing without possible instruction-related attention biases. Performance rates and reaction times were recorded. Prior to scanning, each participant received at least 10 practice trials outside the scanner, which were different from the stimuli used in the main experiment. During the preparation scans additional clips were presented to adjust the volume of the headphone. Each participant performed two runs with 60 video clips and a total duration of 10.5 min each.

### fMRI data acquisition

MRI was performed on a 3T Siemens scanner (Siemens MRT Trio series). Functional data were acquired with echo planar images in 38 transversal slices (repetition time [TR] = 2000 ms; echo time [TE] = 30 ms; flip angle = 90°; slice thickness = 3 mm; interslice gap = 0.30 mm; field of view [FoV] = 220 × 199 mm, voxel resolution = 3.44 × 3.44 mm, matrix dimensions 64 × 58 mm). Slices were positioned to achieve whole brain coverage. During each functional run 315 volumes were acquired.

### Data analysis

MR images were analyzed using Statistical Parametric Mapping (SPM2; www.fil.ion.ucl.ac.uk) implemented in MATLAB 6.5 (Mathworks Inc., Sherborn, MA). The first five volumes of every functional run were discarded from the analysis to minimize T1-saturation effects. To correct for different acquisition times, the signal measured in each slice was shifted relative to the acquisition time of the middle slice using a slice interpolation in time. All images of one session were realigned to the first image of a run to correct for head movement and normalized into standard stereotaxic anatomical MNI-space by using the transformation matrix calculated from the first EPI-scan of each subject and the EPI-template. Afterwards, the normalized data with a resliced voxel size of 3.5 × 3.5 × 3.5 mm were smoothed with a 6 mm FWHM isotropic Gaussian kernel to accommodate intersubject variation in brain anatomy. Proportional scaling with high-pass filtering was used to eliminate confounding effects of differences in global activity within and between subjects.

The expected hemodynamic response at the defined “points of integration” for each event-type was modeled by two response functions, a canonical hemodynamic response function (HRF; Friston et al., 1998) and its temporal derivative. The temporal derivative was included in the model to account for the residual variance resulting from small temporal differences in the onset of the hemodynamic response, which is not explained by the canonical HRF alone. The functions were convolved with the event sequence, with fixed event duration of 1 s, for the onsets corresponding to the integration points of gesture stroke and sentence keyword to create the stimulus conditions in a general linear model (Green et al., 2009; Kircher et al., 2009; Straube et al., 2010, 2011b). The fixed event duration of 1 s was chosen to get a broader range of data around the assumed time point of integration. This methodological approach was also applied successfully in previous studies of co-verbal gesture processing (Kircher et al., 2009; Straube et al., 2010, 2011b).

A group analysis was performed by entering contrast images into a flexible factorial analysis as implemented in SPM5 in which subjects are treated as random variables. A Monte Carlo simulation of the brain volume of the current study was conducted to establish an appropriate voxel contiguity threshold (Slotnick et al., 2003). Assuming an individual voxel type I error of *p* < 0.005, a cluster extent of 8 contiguous re-sampled voxels was necessary to correct for multiple voxel comparisons at *p* < 0.05. Thus, voxels with a significance level of *p* < 0.005 uncorrected, belonging to clusters with at least eight voxels are reported (Straube et al., 2010). Activation peaks of some of the activation clusters also hold a family wise error (FWE) correction. Corresponding corrected *p*-values for each activation peak were included in the tables. The reported voxel coordinates of activation peaks are located in MNI space. Statistical analyses of data other than fMRI were performed using SPSS version 14.0 for Windows (SPSS Inc., Chicago, IL, USA). Greenhouse–Geisser correction was applied whenever necessary.

### Contrasts of interest

To test our hypothesis on the neural processing of different perceptual categories in concrete vs. abstract sentence contexts (cf. Introduction section), baseline contrasts (main effects of condition), conjunction analysis and interaction analysis were run.

At first, baseline contrasts were calculated in order to detect general activations with regard to the four main conditions (aSP, cSP, aSH, cSH) as compared to baseline (fixation cross).

In a next step, main effects (SH vs. SP and a vs. c) as well as the interaction were calculated (t-contrasts) to show brain regions involved in the processing of different factors (directed general effects).

To test the hypothesis that perceptual category is processed in the same neural structures regardless of the language context we performed conjunction analyses of difference contrasts (aSP > aSH ∩ cSP > cSH and aSH > aSP ∩ cSH > cSP). To test for general effects of abstractness independent of both space-related as well as shape-related contents the same approach was used (aSP > cSP ∩ aSH > cSH and cSH > aSH ∩ cSP > aSP).

Finally, we performed two interaction analyses to test the hypothesis that abstractness significantly changes the processing of perceptual categories, space and shape: (1) = (aSP > cSP) > (aSH > cSH) masked for (aSP > cSP) and aSP; (2) = (aSH > cSH) > (aSP > cSP) masked for (aSH > cSH) and aSH. The masking procedure was applied to avoid the interpretation of deactivation in the concrete conditions and restrict the effects to increased activity for aSP vs. low-level baseline and its concrete derivative (cSP). Based on our hypothesis, this methodological approach enables us to find specific neural responses for semantic category (concrete/abstract) in space-related (1) and shape-related (2) perceptual contexts.

## Results

### Behavioral results

The average reaction time for the control task (“indicate the color of the spot on the actor's sweater”) did not differ with regard to color or gesture condition [color: *F*_(1,15)_ = 0.506, *P* = 0.488; condition: *F*_(4,60)_ = 0.604, *P* = 0.604; interaction: *F*_(4,60)_ = 1.256, *P* = 0.301; within-subjects two-factorial ANOVA; mean = 1.23 sec, *SD* = 0.94]. The participants showed an average accuracy rate of 99% which did not differ across conditions [*F*_(4,60)_ = 0.273, *P* = 0.841, within-subjects ANOVA]. Thus, the attention control task indicated that participants did pay attention to the video clips.

### fMRI results

#### Baseline contrasts (aSP, cSP, aSH, cSH)

**Figure 2:**
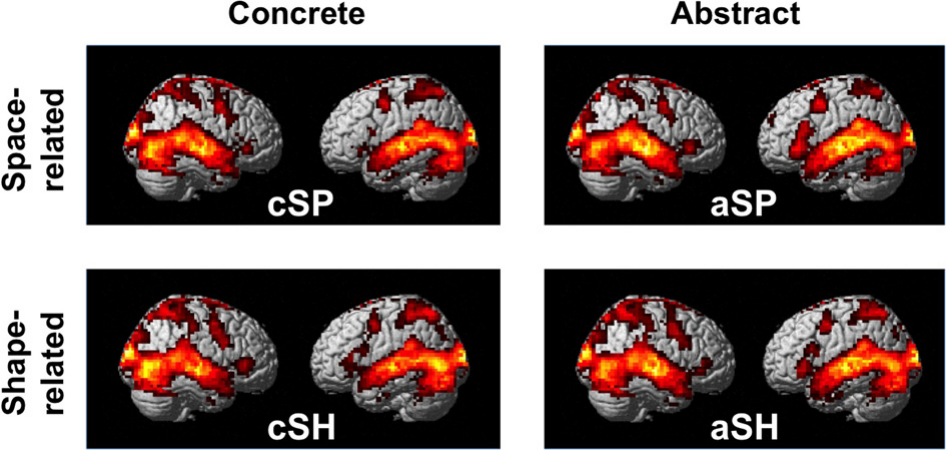
**Activation pattern in contrast to baseline (whole-brain, *p* < 0.005, cluster extend threshold = 8 voxels; MC corrected *p* < 0.05)**.

To explore the general processing mechanisms for each condition and the high comparability between conditions baseline contrasts were calculated (Figure 2, Table 3). We found comparable activation patterns as in previous studies on speech and gesture stimuli (Straube et al., 2011a).

**Table 3:** **Gesture conditions in contrast to low level baseline (fixation cross)**.

**Contrast**	**Anatomical region**	**Hem**.	**BA**	**Coordinates**			***t*-value**	^*^Uncor	^*^FWE	**No. voxels**
				***x***	***y***	***z***				
aSP	Superior temporal gyrus	L	22	−60	−21	0	16.71	< 0.001	< 0.001	7102
	Middle frontal gyrus	L	6	−46	4	53	7.87	< 0.001	< 0.001	127
	Precentral gyrus	R	6	53	0	49	6.57	< 0.001	< 0.001	106
	Superior parietal lobe	L	7	−25	−67	60	4.73	< 0.001	0.224	147
	Cerebellum	R		14	−25	−32	3.75	< 0.001	0.999	10
	Parahippocampal gyrus	L	34	−14	−11	−21	3.75	< 0.001	0.999	19
	Superior frontal gyrus	L	9	−14	53	25	3.68	< 0.001	1.000	20
	Cingulate gyrus	L	31	−11	−46	39	3.50	< 0.001	1.000	10
	Cerebellum	L		−11	−25	−32	3.40	0.001	1.000	15
cSP	Superior temporal gyrus	L	22	−60	−21	0	14.87	< 0.001	< 0.001	7196
	Inferior parietal lobe	L	40	−46	−39	63	6.20	< 0.001	0.001	225
	Caudate (sub-gyral)	L		0	4	18	4.34	< 0.001	0.737	29
	Cingulate gyrus	R	24	21	4	39	4.05	< 0.001	0.950	17
	Amygdala	L		−18	−4	−25	3.92	< 0.001	0.985	17
	Cerebellum	L		−14	−25	−32	3.83	< 0.001	0.995	10
	Precentral gyrus	R		28	−21	42	3.78	< 0.001	0.998	19
aSH	Superior temporal gyrus	L	22	−60	−21	0	16.16	< 0.001	< 0.001	8172
	Precentral gyrus	L	6	−49	0	53	7.03	< 0.001	< 0.001	104
	Middle frontal gyrus	R	6	53	4	49	6.54	< 0.001	< 0.001	182
	Caudate (sub-gyral)	L		−4	−4	21	4.31	< 0.001	0.764	18
	Precuneus	L	7	−25	−74	39	3.96	< 0.001	0.977	23
cSH	Middle occipital gyrus	L	19	−46	−77	0	14.91	< 0.001	< 0.001	7512
	Postcentral gyrus	L	2	−42	−39	63	6.67	< 0.001	< 0.001	371
	Inferior frontal gyrus	R	47	49	35	0	5.39	< 0.001	0.019	76
	Amygdala	L		−18	−4	−25	4.15	< 0.001	0.895	22
	Putamen	L		−21	11	−7	3.06	0.002	1.000	9

*Significance level (t-value), size of the respective activation cluster (No. voxels; number of voxels > 8) at p < 0.005 MC corrected for multiple comparisons. Coordinates are listed in MNI space. BA is the Brodmann area nearest to the coordinate and should be considered approximate. (cSP, concrete spatial; cSH, concrete descriptive; AS, abstract spatial; aSH, abstract descriptive)*.

#### Main effects for perceptual category

To identify the general effect of speech-gesture information, the main effect for the factors perception category [space-related (SP) vs. shape-related (SH)] were calculated.

For the effect of space-related vs. shape-related information (SP > SH) we found an extended network of activations including left middle [Brodmann Area (BA) 6] and superior frontal (BAs 6/8) as well as temporo-parietal (BAs 21/39/40) brain regions (Table 4).

**Table 4:** **Main effects for space-related and shape-related semantic contents**.

**Contrast**	**Anatomical region**	**Hem**.	**BA**	**Coordinates**			***t*-value**	^*^Uncor	^*^FWE	**No. voxels**
				***x***	***y***	***z***				
SP > SH	SMA	L	6	−11	7	74	4.24	< 0.001	0.829	28
	Precentral gyrus	L	44	−42	7	49	3.92	< 0.001	0.986	20
	Inferior parietal lobe	L	39	−46	−81	28	3.81	< 0.001	0.996	10
	Superior frontal gyrus	L	8	−11	39	49	3.73	< 0.001	0.999	21
	Angular gyrus	L	39	−46	−60	32	3.59	< 0.001	1.000	23
	Superior temporal gyrus	L	40	−56	−46	21	3.48	< 0.001	1.000	10
	Middle temporal gyrus	L	21	−53	−21	−15	3.43	0.001	1.000	11
	Fusiform gyrus	R	20	42	−14	−21	3.30	0.001	1.000	9
SH > SP	Inferior occipital gyrus	L	37	−39	−70	−4	5.84	< 0.001	0.003	536
	Superior parietal lobe	R	7	28	−53	56	4.65	< 0.001	0.298	214
	Hippocampus	L		−21	−28	0	4.51	< 0.001	0.497	14
	Inferior frontal gyrus	L	45	−42	35	7	4.50	< 0.001	0.522	10
	Middle orbital gyrus	L	11	−28	39	−14	4.13	< 0.001	0.908	24
	Inferior parietal lobe	L	40	−32	−42	35	3.95	< 0.001	0.979	56
	Inferior frontal gyrus	R	46	46	39	7	3.94	< 0.001	0.982	23
	Middle occipital gyrus	R	17	32	−77	7	3.90	< 0.001	0.988	24
	Precentral gyrus	R	9	53	4	28	3.79	< 0.001	0.997	23
	Inferior occipital gyrus	R	18	28	−88	−11	3.68	< 0.001	1.000	10

*Significance level (*t*-value), size of the respective activation cluster (No. voxels; number of voxels > 8) at *p* < 0.005 MC corrected for multiple comparisons. Coordinates are listed in MNI space. BA is the Brodmann area nearest to the coordinate and should be considered approximate*.

The processing of shape-related vs. space-related information (SH > SP) resulted in enhanced neural responses in bilateral occipital-parietal (BAs 18/37) and middle (BA 11) as well as inferior frontal (BA 45) gyri and left parietal (BA 40) brain region (Table 4).

#### Main effects for abstractness

**Figure 3:**
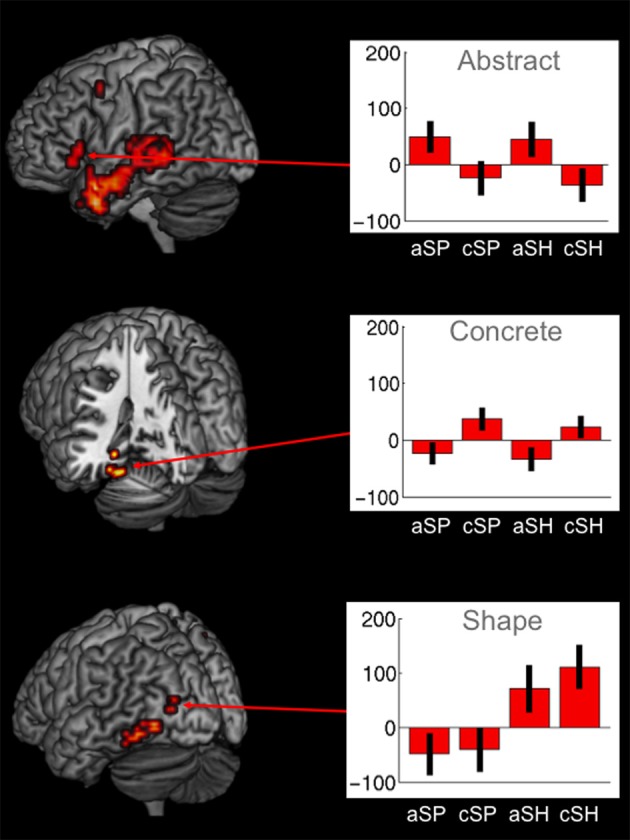
**Significant brain activations for abstractness, concreteness as well as for shape-related co-verbal gesture processing (whole-brain, *p* < 0.005, cluster extend threshold = 8 voxels; MC corrected *p* < 0.05) (cSP, concrete spatial; cSH, concrete shape; AS, abstract spatial; aSH, abstract shape)**.

Abstract vs. concrete speech-gesture information (a > c) revealed a widespread pattern of activation. A large cluster of activation was found in the left IFG extending to the temporal lobe, including the temporal pole and the middle temporal gyrus. Activations were also found in the right superior temporal gyrus, in the left precuneus and right cuneus as well as in the left precentral and superior medial gyri (BAs 6/9). Enhanced neural responses were also found in the middle cingulate, the left superior frontal and superior medial cortex as well as in the left angular gyrus (BA 39/40) (see Table 5, Figure 3).

**Table 5:** **Main effect for abstractness and concreteness**.

**Contrast**	**Anatomical Region**	**Hem**.	**BA**	**Coordinates**			***t*-value**	**Uncor**	**No. voxels**
				***x***	***y***	***z***			
a > c	Temporal pole	L	38	−49	10	−24	7.88	< 0.001	981
	Superior temporal gyrus	R	22	49	−14	0	5.29	< 0.001	313
	Precuneus	L	7	−10	−60	38	4.51	< 0.001	82
	Precentral gyrus	L	9	−38	7	42	4.18	< 0.001	31
	Superior medial gyrus	L	9	−10	60	32	4.05	< 0.001	32
	Cuneus	R	7	21	−60	35	3.90	< 0.001	17
	Superior medial gyrus	L	6	−4	28	60	3.70	< 0.001	10
	Angular gyrus	L	39	−42	−60	32	3.65	< 0.001	28
	Middle cingulate cortex	L	23	−7	−24	32	3.21	0.001	8
c > a	Fusiform gyrus	L	37	−32	−38	−14	5.46	< 0.001	126
	Inferior frontal gyrus	L	46	−42	32	10	4.03	< 0.001	18
	Cerebellum	R		32	−42	−24	3.90	< 0.001	9
	Inferior occipital gyrus	L	37	−49	−70	−7	3.82	< 0.001	25
	Fusiform gyrus	R	36	32	−35	−14	3.81	< 0.001	15
	Middle occipital gyrus	L	19	−35	−88	28	3.70	< 0.001	16
	Middle frontal gyrus	R	11	28	32	−18	3.63	< 0.001	13
	Precentral gyrus	R	4	28	−24	52	3.05	0.002	11

For the reverse contrast (c > a) we found activations in the left and right parahippocampal and fusiform gyri (BA 36/37), in the left inferior frontal (BA 46) and in the temporo-occipital region (BA 37) as well as in the left superior occipital gyrus (BA 19) (see Table 5). Smaller clusters of activation were found in the right cerebellum, the middle frontal (BA 11) and in the precentral gyrus (BA 4).

#### Interaction of perceptual categories and abstractness

For the interaction of perceptual category and abstractness (aSP > cSP)>(aSH > cSH) we found superior medial frontal, left inferior frontal (BA45/44) and middle temporal and superior parietal brain regions (see Table 6).

**Table 6:** **Interaction of semantic categories and abstractness**.

**Contrast**	**Anatomical region**	**Hem**.	**BA**	**Coordinates**			***t*-value**	**Uncor**	**No. voxels**
				***x***	***y***	***z***			
(aSP>cSP)	Superior medial gyrus	L	8	−7	46	52	5.00	< 0.001	36
>	Inferior frontal gyrus	L	47	−52	28	0	4.77	< 0.001	57
(aSH>cSH)	Superior medial gyrus	L	9	−7	49	24	4.27	< 0.001	60
	Inferior frontal gyrus	L	47	−35	28	−18	3.61	< 0.001	8
	Superior parietal lobule	L	7	−28	−66	56	3.52	< 0.001	8
	Middle temporal gyrus	L	22	−60	−46	4	3.07	0.002	11
(aSH>cSH)	Fusiform gyrus	L	19	−28	−46	−10	5.03	< 0.001	48
>	Fusiform gyrus	R	19	28	−49	−10	4.48	< 0.001	23
(aSP>cSP)	Middle frontal gyrus	R	8	38	14	46	4.23	< 0.001	35
	Middle frontal gyrus	R	10	38	52	7	4.16	< 0.001	27
	Calcarine gyrus	R	18	14	−77	4	4.13	< 0.001	81
	Inferior parietal lobule	R	40	49	−49	46	3.67	< 0.001	31
	Inferior frontal gyrus	R	44	46	10	10	3.50	< 0.001	11
	Middle occipital gyrus	R	39	42	−77	32	3.28	0.001	9
	Precuneus	R	7	7	−56	56	3.23	0.001	12
	Paracentral lobule	L	6	−4	−28	52	3.22	0.001	20

For the contrast in the opposite direction (aSH > cSH) > (aSP > cSP) we found a more distributed predominantly right hemispheric activation pattern including the occipital lobe, the middle frontal gyrus, the inferior parietal lobe, the precuneus, the IFG (BA44/45), the middle occipital gyrus and the bilateral fusiform gyri (see Table 6).

#### Specific contrasts of interest

***Brain areas sensitive for shape-related and space-related perceptual contents independent of abstractness***. A conjunction analysis for shape-related form descriptive perceptual contents irrespective of the level of abstractness (aSH > aSP ∩ cSH > cSP) revealed enhanced neural responses in the left middle occipital gyrus (BA 37; see supplementary material Table 7).

No region was found to be significantly activated for space vs. shape-related processing on concrete and abstract level (aSP > aSH ∩ cSP > cSH) (see supplementary material Table 8).

***Brain areas sensitive for abstractness independent of perceptual category (shape/space)***. Common activations for abstract as opposed to concrete co-verbal gestures, irrespective of descriptive or spatial information (aSH > cSH ∩ aSP > cSP), resulted in a large cluster of activation encompassing the left temporal pole and the middle temporal gyrus. Another cluster of activation was found in the right superior temporal gyrus and in the left IFG, including the pars Orbitalis as well as the pars Triangularis (BA 44; see supplementary material Table 9).

The imaging results for concreteness independent of the shape-related or space-related perceptual content (cSH > aSH ∩ cSP > aSP) revealed enhanced BOLD responses in the left parahippocampal gyrus (BA 35; see supplementary material Table 10).

**Figure 4:**
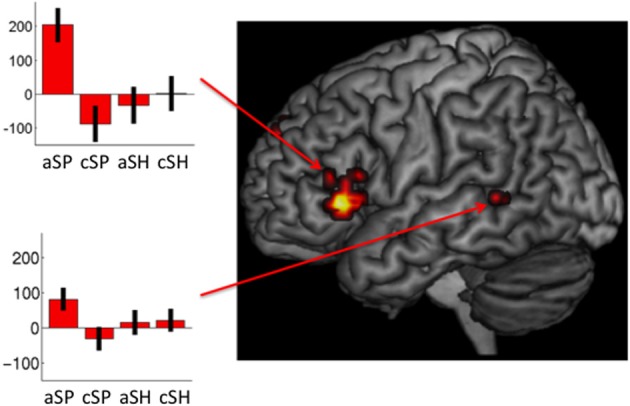
**Interaction of space-related co-verbal information processing and abstractness (whole-brain, *p* < 0.005, cluster extend threshold = 8 voxels; MC corrected *p* < 0.05) (cSP, concrete spatial; cSH, concrete shape; AS, abstract spatial; aSH, abstract shape)**.

***Specific neural responses for abstractness in space-related (1) as well as in shape-related (2) content domains***. The specifically masked interaction analyses (see Contrast of Interest section) revealed distinct activation for abstractness on space-related information [(sSP > cSP) > (aSH > cSH) masked for (aSP > cSP) and aSP] within the left IFG (MNIxyz: −53, 28, 0; *t* = 4.77; 42 voxels) and the left pTL (MNIxyz: −60, −46, 4; *t* = 3.07; 10 voxels; see Figure 4). The other direction of contrasts did not reveal any significant results.

Taken together, significant main effects and interactions of brain activation with regard to the manipulated factors [type of communicated perceptual information (SP, SH) and abstractness (c, a)] revealed different patterns of activation. The specific contrasts indicated that subregions of the left IFG and the left pTL have common [conjunction analyses: IFG [MNIxyz: −39, 28, −4; *t* = 3.27; 11 voxels], pTL (MNIxyz: −53, −38, 0; *t* = 4.26; 196)] and distinct functions [interaction: IFG (MNIxyz: −53, 28, 0; *t* = 4.77; 42 voxels], pTL [MNIxyz: −60, −46, 4; *t* = 3.07; 10 voxels)] with regard to perceptual type and abstractness.

The same analysis, including only right-handed gesture stimuli of equal length (speech duration) revealed the same pattern of activation encompassing the left IFG as well as the left middle temporal gyrus, indicating that this effect is not based on irrelevant differences in stimulus material.

## Discussion

Space and shape are distinct perceptual categories. Words referring to space and shape also describe abstract concepts like “rising income” (space) or a “square personality” (shape). Gestures are an important part of human communication that underpin verbal utterances and can convey shape or space information even when accompanying abstract sentences. Recent studies have investigated the neural processing of speech and gesture (Willems and Hagoort, 2007; Willems et al., 2007, 2009; Dick et al., 2009, 2012; Green et al., 2009; Hubbard et al., 2009; Kelly et al., 2010; Kircher et al., 2009; Skipper et al., 2009; Straube et al., 2009; Holle et al., 2010). Despite the fact that the investigation of perceptual categories used in speech and gesture could give important answers with regard to the effect of abstractness on particular neural networks relevant for the processing of such perceptual information, the related effect is not known. Thus, the purpose of the current fMRI study was to investigate the neural processing of shape-related vs. space-related co-speech gesture information when presented with abstract or concrete utterances aiming at the question whether similar or distinct neural networks are involved.

In line with previous findings (Straube et al., 2011a) we found enhanced cortical activations for abstract (a) as opposed to concrete (c) utterances in the bilateral temporal lobes and in the left IFG for both, space as well as shape-related sentences (aSP > cSP and aSH > cSH). The interaction of perceptual category and abstractness in a more anterior part of the left IFG and inferior part of the pTL indicates that abstractness strongly influenced the neural processing of space and shape information. Only the effect of shape- vs. space-related information revealed activation in a single cluster of the left inferior occipital gyrus independent of abstractness (cSH > cSP ∩ aSH c> aSP). By contrast, the interaction resulted in enhanced BOLD responses in a more anterior part of the left IFG and inferior part of the pTL. Thus, we demonstrate the interaction of perceptual category and abstractness on the neural processing of speech accompanied by gestures. These data suggest a functional division of the pTL and left IFG being sensitive to the processing of both the level of abstractness and the type of categorical information. These imaging results further offer neural support for the traditional categorization of co-verbal gestures with regard to their content and abstractness (McNeill, 1992, 2005).

The imaging results for the abstract co-verbal gesture condition revealed BOLD enhancements in the left inferior frontal and the bilateral temporal regions, respectively. This finding is consistent with previous evidence of involvement of the left IFG and bilateral temporal lobes in the integration of gestures with abstract sentences (Kircher et al., 2009; Straube et al., 2009, 2011a). With regard to the underlying neuro-cognitive processes, we assume that the concrete visual gesture information (e.g., illustrating an arch of a bridge) is being interpreted in context of the abstract sentence meaning (“the politician builds a bridge to the next topic”). Thus, correspondence of gesture and sentence meaning must be identified and figurative components of speech and gesture must be translated from their literal/concrete meanings. To build this relation between speech and gesture information on the level of abstractness, additional online unification processes within the IFG seem to be relevant (Straube et al., 2011a). Such processes might be similar to those responsible for making inferences (e.g., Bunge et al., 2009, relational reasoning (e.g., Wendelken et al., 2008), the building of analogies (e.g., Luo et al., 2003; Bunge et al., 2005; Green et al., 2006; Watson and Chatterjee, 2012), and unification (Hagoort et al., 2009; Straube et al., 2011a). Those processes may also be involved in the comprehension of novel metaphoric or ambiguous communications and consistently activate the left IFG (Rapp et al., 2004, 2007; Stringaris et al., 2007; Chen et al., 2008; Cardillo et al., 2012). Consequently, enhanced neural responses in the fronto-temporal network may be evoked by the higher cognitive demand in an abstract metaphoric context which may have resulted in the recruitment of the left inferior frontal and middle temporal region (Kircher et al., 2009; Straube et al., 2011a).

Concrete speech accompanied by gestures revealed a pattern of enhanced BOLD responses in parahippocampal regions bilaterally as well as in the left superior occipital gyrus. Concrete co-verbal utterances such as, “the workman builds a bridge over the river,” evokes a comparatively transparent connection/relation to a familiar everyday event. Accordingly, an experienced-based understanding of a scene may have resulted in the recruitment of the parahippocampal regions, whereas the direct imagery of concrete objects or actions may have resulted in enhanced neural responses in the left superior occipital region (Green et al., 2009) facilitating the understanding of the concrete co-verbal content.

The shape-related sentences accompanied by shape-related gestures revealed activations in the left middle occipital region. Similar to the activations found for the concrete condition (c > a), imagery of an experience-based perceptual representation resulted in the activations of the left occipital area. However, we did not observe common activation for the processing of spatial information in a concrete and abstract sentence context. Together these data do not support a universal neural processing of space and shape in a multimodal communication context.

By contrast, we found an interaction for perceptual category and abstractness, as spatial information on an abstract level (aSP) specifically (in contrast to all other conditions) activated a particular part of the left IFG and the left superior temporal region. This finding was robust and independent of both hand movement and speech duration. Thus, BOLD enhancements in these regions suggest that predominantly spatial information is processed differently in an abstract vs. concrete sentence context. Additional semantic information is retrieved from the left superior temporal region. The higher cognitive load together with the resulting enhanced effort with regard to information-specific abstract and spatial lexical retrieval may account for the recruitment of the fronto-temporal network. However, specific activation of the left IFG could also represent competition between meanings of spatial terms in the aSP condition, including at a minimum the concrete/literal and the abstract/metaphoric interpretations (Chatterjee, 2008; Chen et al., 2008).

For the processing of shape-related information we found common activation within the inferior temporal gyrus and the occipital lobe for concrete and abstract utterances, suggesting a common perceptual representation activated during comprehension of shape information. This perceptual representation probably compensated for the need of additional resources of the IFG and pTL, which were activated for space-related information in an abstract sentence context. Thus, this finding suggests that a concrete representation of shape is also activated in an abstract sentence context. This might have further facilitated the processing of the abstract representation of shape. For the processing of space-related information we found no common activation for concrete and abstract utterances, indicating different neural processing mechanism for both types of communications. The transformation of space-related gesture information in an abstract sentence context probably required higher order semantic processing mechanisms (Straube et al., 2011a) which probably inhibited the actual perceptual spatial representation of these gestures.

A limitation of this study is that the specific effects of gesture as well as integration processes cannot be disentangled. Distinguishing between speech and gesture was not the purpose of the current study. The problem with regard to the interpretation of our results for the main effect of abstractness, irrespective of perceptual category, might be that the activation patterns found for abstract speech accompanied by gestures in the left IFG and bilateral temporal lobes is produced by differences in the abstractness between the sentences, as demonstrated by several studies about metaphoric speech processing (Rapp et al., 2004, 2007; Eviatar and Just, 2006; Mashal et al., 2007, 2009; Nagels et al., 2013; Stringaris et al., 2007; Chen et al., 2008). However, in a previous study we observed increased activation in the left IFG for metaphoric co-verbal gestures in contrast to control sentences with the identical abstract semantic content (Kircher et al., 2009). Furthermore, there is evidence that activation of the left IFG is specifically related to the processing of novel and therefore unconventional metaphoric sentences (Rapp et al., 2004, 2007; Cardillo et al., 2012), in which abstract information must be interpreted online in terms of its non-literal meaning. However, the abstract sentences used in the current study were conventional and part of everyday communication, e.g., “The talk was on a high level.” This is supported by our rating results, which revealed no differences between the conditions with regard to familiarity. Despite the fact, that we cannot exclude that differences between conditions might be explained by differences in difficulty due to our language manipulation (concrete vs. abstract), the lack of commonalities (e.g., Spa > SHa ∩ SPc > SHc) cannot be explained by these potential differences. The robustness of the imaging results in the aforementioned regions is further supported by the separate control analyses encompassing a carefully matched subset of paired (hand movements and speech length) stimuli.

A further limitation is that the distinction between space- and shape-related information in the current experiment is artificial and do not represent independent factors. Shape gestures include some spatial information. However, despite this intrinsic connection between space and shape, our data demonstrate that these perceptual categories can be distinguished by independent raters and produce distinct interacting activation patterns with regard to abstractness. Therefore, our data support the validity of this separation, which has been traditionally applied in terms of deictic or abstract deictic gestures (which refer to space) in contrast to iconic and metaphoric gestures (which rather refer to form or shape; e.g., McNeill, 1992).

With this study we demonstrate the interaction of perceptual category and abstractness in the neural processing of speech-gesture utterances. Besides abstractness, the type of information was relevant to the neural processing of speech accompanied by gestures. This finding illustrates the relevance of the interaction between language and cognition, which characterizes the complexity of natural interpersonal communication. Future studies should therefore consider the importance of perceptual type and abstractness for the interpretation of their imaging results. Our data suggest a functional subdivision of the pTL and left IFG with regard to the processing of space and shape-related information in an abstract sentence context. Such differences support the theoretically based traditional categorization of co-verbal gestures with regard to information type and abstractness (McNeill, 1992). Most likely the investigation of other types of co-verbal gestures will demonstrate further important differences in the processing of specific co-verbal gesture types, which will enlighten the fine-grained differences of processing mechanisms, which underlie the comprehension of multimodal natural communication.

### Conflict of interest statement

The authors declare that the research was conducted in the absence of any commercial or financial relationships that could be construed as a potential conflict of interest.

## Supplementary material

Table 7 **Brain areas sensitive for shape-related contents (independent of abstractness)**. Significance level (t-value), size of the respective activation cluster (No. voxels; number of voxels > 8) at p < 0.005 MC corrected for multiple comparisons. Coordinates are listed in MNI space. BA is the Brodmann area nearest to the coordinate and should be considered approximate. (cSP, concrete spatial; cSH, concrete shape; aSP, abstract spatial; aSH, abstract shape).

Table 8 **Brain areas sensitive for space-related contents (independent of abstractness)**. Significance level (t-value), size of the respective activation cluster (No. voxels; number of voxels > 8) at p < 0.005 MC corrected for multiple comparisons. Coordinates are listed in MNI space. BA is the Brodmann area nearest to the coordinate and should be considered approximate. (cSP, concrete spatial; cSH, concrete shape; aSP, abstract spatial; aSH, abstract shape).

Table 9 **Brain areas sensitive for abstractness (independent of content)**. Significance level (t-value), size of the respective activation cluster (No. voxels; number of voxels > 8) at p < 0.005 MC corrected for multiple comparisons. Coordinates are listed in MNI space. BA is the Brodmann area nearest to the coordinate and should be considered approximate. (cSP, concrete spatial; cSH, concrete shape; aSP, abstract spatial; aSH, abstract shape).

Table 10 **Brain areas sensitive for concreteness (independent of content)**. Significance level (t-value), size of the respective activation cluster (No. voxels; number of voxels > 8) at p < 0.005 MC corrected for multiple comparisons. Coordinates are listed in MNI space. BA is the Brodmann area nearest to the coordinate and should be considered approximate. (cSP, concrete spatial; cSH, concrete shape; aSP, abstract spatial; aSH, abstract shape).
