# Supplementary material for: The role of semantic abstractness and perceptual category in processing speech accompanied by gestures
Source: Front Behav Neurosci. 2013 Dec 18;7:181. doi: 10.3389/fnbeh.2013.00181 (PMC3866656; doi:10.3389/fnbeh.2013.00181)
Supplement: Table 7 — Brain areas sensitive for shape-related contents (independent of abstractness). Significance level (t-value), size of the respective activation cluster (No. voxels; number of voxels > 8) at p < 0.005 MC corrected for multiple comparisons. Coordinates are listed in MNI space. BA is the Brodmann area nearest to the coordinate and should be considered approximate. (cSP, concrete spatial; cSH, concrete shape; aSP, abstract spatial; aSH, abstract shape). [file DataSheet1.PDF]

Supplementary Material

Table 7: Brain areas sensitive for shape-related contents (independent of abstractness)

Contrast	Anatomical Region	Hem.	BA	Coordinates			t-value	uncor	No. voxels
				x	y	z			
**aSH > aSP**	Cerebellum	L		-28	-49	-18	4.36	< .001	82
	Inferior Parietal Lobe	R	40	46	-46	46	4.23	<.001	147
	Cerebellum	R		7	-46	-7	3.98	<.001	33
	Middle Frontal Gyrus	R	46	49	42	21	3.88	<.001	73
	Cingulate Gyrus	L	24	-4	4	32	3.67	<.001	8
	Middle Temporal Gyrus	L	19	-35	-84	25	3.56	<.001	20
	Middle Occipital Gyrus	L	37	-49	-67	-11	3.56	<.001	13
	Inferior Parietal Lobe	L	40	-32	-53	56	3.53	<.001	16
	Superior Frontal Gyrus	L	11	-32	46	-14	3.44	0.001	10
	Parietal Lobe (sub-gyral)	L		-28	-42	35	3.00	0.002	10
**cSH > cSP**	Fusiform Gyrus	L	37	-46	-53	-14	4.63	<.001	230
	Superior Parietal Lobe	L	7	-28	-67	56	4.38	<.001	97
	Inferior Parietal Lobe	L	40	-53	-32	39	4.12	<.001	25
	Precuneus	L	7	-28	-46	39	3.99	<.001	8
	Middle Occipital Gyrus	R	19	28	-81	7	3.95	<.001	22
	Inferior Frontal Gyrus	R	9	53	4	28	3.95	<.001	22
	Inferior Frontal Gyrus	L	46	-46	32	14	3.86	<.001	38
	Inferior Frontal Gyrus	R	46	49	39	4	3.82	<.001	13
	Inferior Frontal Gyrus	L	9	-49	4	32	3.81	<.001	22
	Parietal Lobe (sub-gyral)	R	7	25	-53	56	3.71	<.001	24
	Inferior Parietal Lobe	R	40	32	-42	42	3.61	<.001	20
	Superior Frontal Gyrus	R	9	14	49	28	3.51	<.001	9
	Caudate	L		-14	-4	32	3.43	0.001	10
	Postcentral Gyrus	R	3	56	-25	39	3.38	0.001	10
	Medial Frontal Gyrus	L	9	-11	46	28	3.37	0.001	12
**Conj.**	Middle Occipital Gyrus	L	37	-39	-70	-4	3.84	<.001	13
(aSH>aSP)∩ (cSH>cSP)	Middle Occipital Gyrus	L	37	-49	-67	-11	3.56	<.001	10

Significance level (t-value), size of the respective activation cluster (No. voxels; number of voxels > 8) at p < .005 MC corrected for multiple comparisons. Coordinates are listed in MNI space. BA is the Brodmann area nearest to the coordinate and should be considered approximate. (cSP = concrete spatial; cSH = concrete shape; aSP = abstract spatial; aSH = abstract shape)

Table 8: Brain areas sensitive for space-related contents (independent of abstractness)

Contrast	Anatomical Region	Hem.	BA	Coordinates x	y	z	t-value	uncor	No. voxels
**aSP > aSH**	Superior Frontal Gyrus	L	8	-7	46	53	5.68	<.001	107
	Inferior Frontal Gyrus	L	47	-53	28	0	4.84	<.001	53
	Superior Temporal Gyrus	L	39	-46	-60	32	4.54	<.001	60
	Cerebellum	L		-11	-28	-21	4.14	<.001	8
	Superior Frontal Gyrus	L	6	-4	11	67	4.06	<.001	38
	Cerebellum	R		7	-39	-28	4.00	<.001	11
	Cerebellum	R		14	-28	-21	3.89	<.001	9
	Inferior Parietal Lobe	L	40	-60	-46	21	3.73	<.001	9
	Superior Temporal Gyrus	L	22	-56	-42	4	3.64	<.001	60
	Middle Frontal Gyrus	L	6	-42	4	49	3.34	0.001	14
**cSP > cSH**	Middle Frontal Gyrus	R	8	39	14	46	4.77	<.001	32
	Lingual Gyrus	R	18	11	-77	4	4.66	<.001	108
	Parahippocampal Gyrus	L	19	-32	-46	-7	4.01	<.001	13
	Superior Occipital Gyrus	L	19	-42	-81	32	3.93	<.001	14
	Angular Gyrus	R	39	46	-77	32	3.74	<.001	12
	Precuneus	R	7	7	-56	56	3.73	<.001	10
	Temporal Lobe (sub-gyral)	R		28	-56	11	3.43	0.001	10
	Insula	R	13	35	14	18	3.34	0.001	12
**Conj** *(aSP>aSH)∩ (cSP>cSH)*								n.s.	

Significance level (t-value), size of the respective activation cluster (No. voxels; number of voxels > 8) at p < .005 MC corrected for multiple comparisons. Coordinates are listed in MNI space. BA is the Brodmann area nearest to the coordinate and should be considered approximate. (cSP = concrete spatial; cSH = concrete shape; aSP = abstract spatial; aSH = abstract shape)

Table 9: Brain areas sensitive for abstractness (independent of content)

Contrast	Anatomical Region	Hem.	BA	Coordinates x	y	z	t-value	uncor	FWE	No. voxels
**aSP > cSP**	Middle Temporal Gyrus	L	21	-56	-11	-11	6.79	<.001	<.001	771
	Superior Frontal Gyrus	L	9	-7	53	35	5.17	<.001	0.045	134
	Superior Temporal Gyrus	R	22	49	-14	0	4.38	<.001	0.702	146
	Inferior Parietal Lobe	L	40	-35	-60	39	4.12	<.001	0.918	62

	Superior Frontal Gyrus	L	6	-4	14	67	3.93	<.001	0.984	14
	Cingulate Gyrus	L	31	-11	-49	39	3.83	<.001	0.995	59
	Cerebellum	R		21	-74	-32	3.80	<.001	0.997	20
	Superior Temporal Gyrus	R	38	49	11	-18	3.64	<.001	1.000	35
**aSH > cSH**	Superior Temporal Gyrus	L	38	-49	11	-25	6.40	<.001	<.001	398
	Precuneus	L	7	-7	-67	39	4.65	<.001	0.302	95
	Superior Temporal Gyrus	R	22	49	-28	0	4.26	<.001	0.818	118
	Middle Frontal Gyrus	R	8	39	14	46	3.99	<.001	0.971	12
	Middle Frontal Gyrus	R	10	42	53	14	3.72	<.001	0.999	15
	Cingulate Gyrus	L	24	0	-11	42	3.72	<.001	0.999	18
	Inferior Frontal Gyrus	L	47	-39	28	-4	3.27	0.001	1.000	11
**Conj** *(aSH>cSH)∩ (aSP>cSP)*	Temporal Pole/ Middle Temporal Gyrus	L	22/23	-49	11	-25	5.45	<.001	0.015	196
	Superior Temporal Gyrus	R	21/22	53	-28	7	3.74	<.001	0.999	75
	Inferior Frontal Gyrus	L	44/45	-39	28	-4	3.27	0.001	1.000	11

Significance level (t-value), size of the respective activation cluster (No. voxels; number of voxels > 8) at p < .005 MC corrected for multiple comparisons. Coordinates are listed in MNI space. BA is the Brodmann area nearest to the coordinate and should be considered approximate. (cSP = concrete spatial; cSH = concrete shape; aSP = abstract spatial; aSH = abstract shape)

Table 10: Brain areas sensitive for concreteness (independent of content)

				**Coordinates**						
**Contrast**	**Anatomical Region**	**Hem.**	**BA**	**x**	**y**	**z**	**t-value**	**uncor**	**FWE**	**No. voxels**
**cSP > aSP**	Fusiform Gyrus	L	37	-32	-46	-11	6.70	<.001	<.001	174
	Parahippocampal Gyrus	R	37	32	-42	-11	4.29	<.001	0.783	60
	Superior Occipital Gyrus	L	39	-32	-81	28	4.00	<.001	0.966	25
	Postcentral Gyrus	L	3	-28	-28	49	3.85	<.001	0.994	10
	Lingual Gyrus	R	19	14	-49	-4	3.75	<.001	0.998	17
	Lingual Gyrus	R	18	11	-77	0	3.72	<.001	0.999	40
	Middle Frontal Gyrus	R	11	21	28	-14	3.55	<.001	1.000	12
	Lingual Gyrus	L	18	-11	-70	-7	3.44	0.001	1.000	12
	Precentral Gyrus	R	4	28	-25	53	3.35	0.001	1.000	9
	Cuneus	L	17	-14	-84	4	3.23	0.001	1.000	15
	Superior Parietal Lobe	L	5	0	-42	60	3.15	0.001	1.000	9
**cSH > aSH**	Inferior Frontal Gyrus	L	46	-42	32	14	5.45	<.001	0.015	22
	Superior Parietal Lobe	L	7	-28	-67	56	4.21	<.001	0.859	9
	Inferior Temporal Gyrus	L	19	-46	-60	-7	3.94	<.001	0.981	41

	Middle Frontal Gyrus	L	46	-46	35	28	3.85	<.001	0.994	13
	Cerebellum	L		-28	-35	-25	3.50	<.001	1.000	12
	Caudate (sub-lobar)			11	14	4	3.37	0.001	1.000	10
**Conj** *(cSH>aSH)∩ (cSP<aSP)*	Parahippocampal Gyrus	L	35	-28	-28	-25	3.41	0.001	1.000	10

Significance level (t-value), size of the respective activation cluster (No. voxels; number of voxels > 8) at p < .005 MC corrected for multiple comparisons. Coordinates are listed in MNI space. BA is the Brodmann area nearest to the coordinate and should be considered approximate. (cSP = concrete spatial; cSH = concrete shape; aSP = abstract spatial; aSH = abstract shape)
